# Retroaortic Left Renal Vein in a Case of Left Adrenal Adenoma: Radiological Findings

**DOI:** 10.1155/2011/867895

**Published:** 2011-03-22

**Authors:** Alper Dilli, Umit Yasar Ayaz, Osman Raif Karabacak, Baki Hekimoglu

**Affiliations:** ^1^Department of Radiology, Dışkapı Yıldırım Beyazıt Training and Research Hospital, Ministry of Health, Irfan Bastug Street, Diskapi, Altındağ, 06110 Ankara, Turkey; ^2^Department of Radiology, Mersin Women's and Children's Hospital, Ministry of Health, Halkkent, 33240 Mersin, Turkey; ^3^Department of Urology, Dışkapı Yıldırım Beyazıt Training and Research Hospital, Ministry of Health, Irfan Bastug Street, Diskapi, Altındağ, 06110 Ankara, Turkey

## Abstract

It is important to diagnose retroaortic left renal vein (RLRV) before a probable retroperitoneal surgery in a case of a suspicious adrenal mass. Our purpose is to present the ultrasonography (US), computed tomography (CT), and magnetic resonance imaging (MRI) findings in a case of left adrenal adenoma with a coincidental RLRV and to discuss the clinical importance of their imaging. Abdominal and scrotal US, abdominal CT and MRI were performed for a 50-year-old male patient who was referred with continuous abdominal pain, intractable hypertension, high levels of blood cortisol and proteinuria. On US, a hypoechoic solid mass measuring 4 × 3 cm in the left adrenal location and coincidental RLRV, besides multiple renal cysts, hepatomegaly, left-sided varicocele, and small-sized left testis were detected. CT and MRI also revealed the mass in the left adrenal gland which was consistent with adenoma. With CT and MRI, presence of RLRV was also verified.

## 1. Introduction

Left renal vein which has a more complicated embryological development process than the right renal vein may also show significant variations anatomically. Retroaortic left renal vein (RLRV) and circumaortic left renal vein are the most common left renal variations. Satyapal et al. and Yeşildağ et al. found the incidence of RLRV as 0.5% and 2.3%, respectively [1, 2]. Adrenocortical adenomas are benign neoplasms of the adrenal cortex and may be secretory or non-secretory. In about 2% of all cross-sectional imaging, adrenal mass could be demonstrated [3], where this may increase up to 9% in autopsy series [4]. These data about the incidences make it clear that the coexistence of RLRV and an adrenal mass, like an adenoma, is a rare entity. Variations of left renal vein are important to be determined radiologically prior to retroperitoneal surgery. Our purpose is to present the radiological findings in a case of left adrenal adenoma with a coincidental RLRV and to discuss the clinical importance of their imaging. 

## 2. Case Report

A fifty-year-old man was referred with continuous abdominal pain and intractable hypertension. Physical examination revealed hepatomegaly. In laboratory study, high levels of blood cortisol and proteinuria were detected. Abdominal and scrotal ultrasonography (US), abdominal computed tomography (CT), and magnetic resonance imaging (MRI) were performed. All the procedures were performed according to the World Medical Association Declaration of Helsinki. The patient was informed about the imaging procedures, and informed consent was obtained from him. In-phase, out-of-phase sequences and magnetic resonance spectroscopy (MRS) were obtained beside routine MRI sequences.

**Figure 1:**
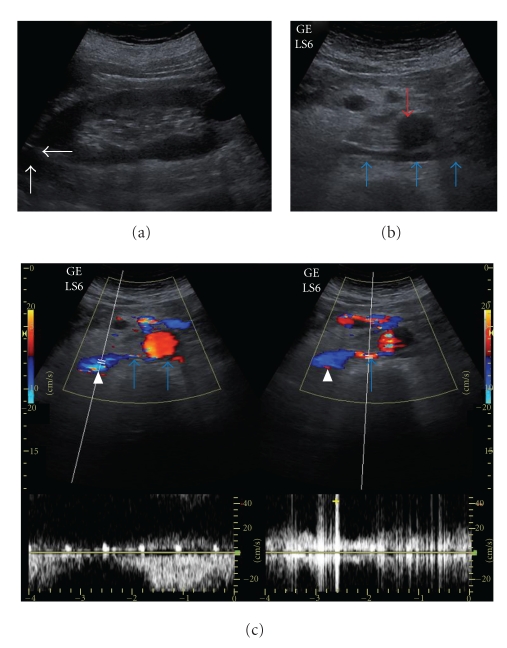
Ultrasonography (US) shows a hypoechoic solid mass in left adrenal location in longitudinal view (white arrows) (a). In transverse images, US demonstrates retroaortic left renal vein (RLRV) (blue arrows), originating from left renal hilus, passing posteriorly to the abdominal aorta (red arrow) and anteriorly to the vertebral corpus, and joining inferior vena cava (white arrowhead) (b). Color Doppler demonstrates RLRV (blue arrows) (c).

**Figure 2:**
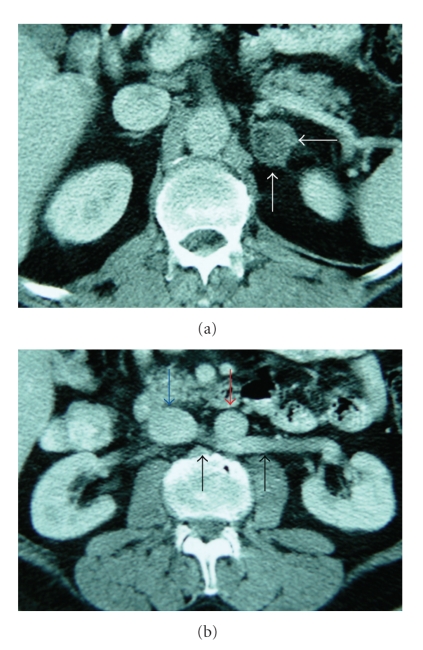
Axial contrast-enhanced computed tomography (CT) images revealed hypodense mass measuring 3.5 × 3 cm within the left adrenal gland (white arrows). The density of the mass was +14 HU after 65 seconds following contrast administration (a). RLRV (black arrows) passing posteriorly to the abdominal aorta (red arrow), joining inferior vena cava (blue arrow), was also depicted (b).

**Figure 3:**
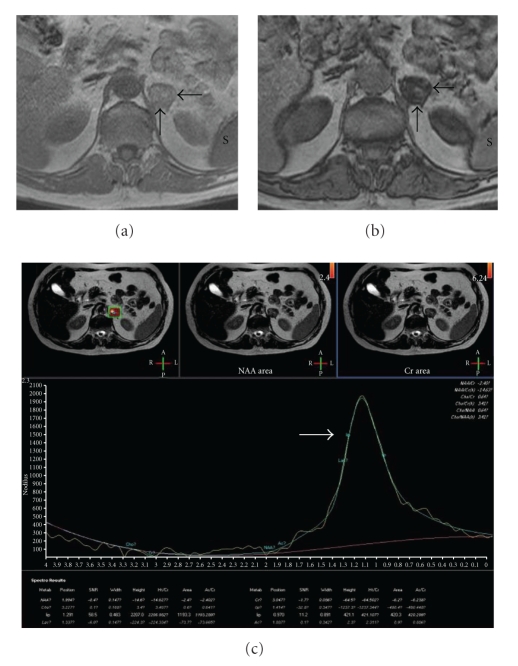
In axial magnetic resonance (MR) imaging, in-phase (a) and out-of-phase (b) images demonstrate a prominent signal loss in the adrenal mass (black arrows) as we take the intensity of the spleen (S) as an internal standard. MR spectroscopy (c) revealed the presence of high lipid content in the left adrenal mass (white arrow). All these findings were consistent with adrenal adenoma.

In US, a hypoechoic solid mass measuring 4 × 3 cm was detected in the left adrenal location. Multiple renal cysts, hepatomegaly with a maximum craniocaudal size of 20 cm, left-sided grade 1 varicocele, and small-sized left testis were other abnormal US findings. As an incidental finding, a venous structure, originating from the left renal hilus, passing posteriorly to the abdominal aorta, and draining to the inferior vena cava was demonstrated and evaluated as RLRV (Figures 1(a), 1(b), 1(c)). Nonenhanced CT revealed a hypodense oval mass measuring 3.5 × 3 cm in the left adrenal gland. The density of the mass was −20 Hounsfield Unit (HU) before contrast administration, whereas the density values were +14 HU and −10 HU after 65 seconds and 15 minutes following contrast administration, respectively. The percentage of washout was calculated to be above 70%, which was consistent with an adenoma. RLRV was depicted with CT (Figures 2(a), 2(b)). In MRI, in-phase, out-of-phase images and MRS revealed the presence of lipid in the left adrenal mass, which was consistent with adrenal adenoma (Figures 3(a), 3(b), 3(c)). RLRV was demonstrated with MRI. After the completion of radiological examinations, the referring surgeons were informed about RLRV. The patient has been in clinical followup since then. 

## 3. Discussion

In the embryologic period, generally the anterior part of the circumaortic venous plexus continues to exist as a normal left renal vein. The absence of the anterior part with persistence of posterior component causes formation of RLRV which crosses the aorta posteriorly [1, 5]. To display anomalies regarding left renal vein, radiological modalities such as US, colour Doppler US, angiography, MRI, and CT can be used [2, 6]. US and colour Doppler US modalities may be preferred because of their being relatively low-cost and noninvasive, but they may be insufficient in overweight patients. The diagnosis of renal vein anomalies is important in retroperitoneal surgery. Unawareness of this situation during retroperitoneal surgery can result in bleeding, nephrectomy, and even death [7]. Furthermore, hematuria, proteinuria, or varicocele may be caused by left renal vein anomalies which are usually detected incidentally [8–11]. Because of this, left renal vein anomalies should be considered in differential diagnosis of the etiology of these signs. In such patients, radiological diagnosis of left renal vein anomalies is important in terms of treatment and exclusion of other etiologies. In our case, proteinuria and left-sided varicocele were associated with RLRV.

Although adrenal adenomas are generally less than 2 cm in diameter and are nonsecretory, they may be larger and have secretory characteristics, as it was considered to be in our case. In our patient, in whom surgery was a possibility initially because of the large suspicious left adrenal mass, RLRV was demonstrated clearly.

In conclusion, RLRV, which is an important vascular variation and the detection of which is crucial to avoid the complication of catastrophic hemorrhage before aortic, renal, and retroperitoneal surgery, could be demonstrated in our case, as well as a relatively large left adrenal adenoma, by all radiological modalities mentioned above. Surgeons were informed about the coexistence of these two entities.
